# Transformable liquid-metal nanomedicine

**DOI:** 10.1038/ncomms10066

**Published:** 2015-12-02

**Authors:** Yue Lu, Quanyin Hu, Yiliang Lin, Dennis B. Pacardo, Chao Wang, Wujin Sun, Frances S. Ligler, Michael D. Dickey, Zhen Gu

**Affiliations:** 1 Joint Department of Biomedical Engineering, University of North Carolina at Chapel Hill and North Carolina State University, Raleigh, North Carolina 27695, USA; 2 Division of Molecular Pharmaceutics and Center for Nanotechnology in Drug Delivery, Eshelman School of Pharmacy, University of North Carolina at Chapel Hill, Chapel Hill, North Carolina 27599, USA; 3 Department of Chemical and Biomolecular Engineering, North Carolina State University, Raleigh, North Carolina 27695, USA; 4 Department of Medicine, University of North Carolina at Chapel Hill, Chapel Hill, North Carolina 27599, USA

## Abstract

To date, numerous inorganic nanocarriers have been explored for drug delivery systems (DDSs). However, the clinical application of inorganic formulations has often been hindered by their toxicity and failure to biodegrade. We describe here a transformable liquid-metal nanomedicine, based on a core–shell nanosphere composed of a liquid-phase eutectic gallium-indium core and a thiolated polymeric shell. This formulation can be simply produced through a sonication-mediated method with bioconjugation flexibility. The resulting nanoparticles loaded with doxorubicin (Dox) have an average diameter of 107 nm and demonstrate the capability to fuse and subsequently degrade under a mildly acidic condition, which facilitates release of Dox in acidic endosomes after cellular internalization. Equipped with hyaluronic acid, a tumour-targeting ligand, this formulation displays enhanced chemotherapeutic inhibition towards the xenograft tumour-bearing mice. This liquid metal-based DDS with fusible and degradable behaviour under physiological conditions provides a new strategy for engineering theranostic agents with low toxicity.

## Abstract (web summary)

The use of inorganic carriers for drug delivery is often limited by toxicity and persistence of inorganic species in the body. Here, the authors report the use of nanocarriers with a liquid-phase eutectic gallium-indium core capable of delivering doxorubicin and subsequently fusing and degrading under mildly acidic conditions.

## Graphical abstract

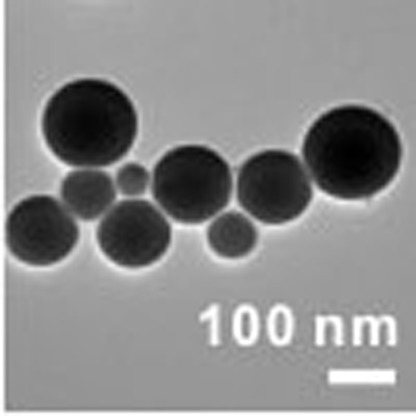

The engineering flexibility provided by inorganic nanoparticles with tailorable shape, size, surface ligands and physical properties has enabled on-demand design of novel drug delivery systems, contrast agents and integrated systems for disease diagnosis and treatment^1^,^2^,^3^,^4^,^5^,^6^. The last decade has witnessed numerous efforts in developing inorganic nanoparticles capable of effectively targeting different diseases^7^,^8^,^9^,^10^,^11^. However, these formulations often fail to be useable due to systemic toxicity. For instance, targeted cancer therapy requires nanoparticles with relatively large sizes to minimize clearance^12^ and enhance tumour retention^13^,^14^, yet such inorganic particles often remain in the body for a long time because of their lack of biodegradability. To date, few studies have demonstrated how to engineer the physicochemical properties of inorganic nanoparticles to satisfy both target delivery and efficient elimination^15^,^16^. This design bottleneck has long existed and is impeding the clinical translation of therapy and diagnostics based on inorganic carriers.

We report here a novel liquid metal-based nanoscale formulation for drug delivery to achieve enhanced anticancer therapy. Taking advantage of the unique characteristics of a eutectic alloy of gallium indium (EGaIn, 75% Ga and 25% In by weight), this novel nanomedicine has a variety of merits in terms of simple fabrication, facile surface bioconjugation and the capability of fusion and degradation in a mildly acidic environment. EGaIn is a low-viscosity liquid at room temperature. Unlike Hg, it has low toxicity and has thus attracted considerable attention for applications of microfluidic systems, soft robotics and stretchable electronics^17^,^18^,^19^. To obtain EGaIn-based drug nanocarriers, an emulsion-like ligand-mediated procedure is simply applied through ultrasonication at room temperature. During sonication^20^, the thiolated ligands readily assemble onto the surface of the EGaIn, competing with the oxidation process and facilitating control of the particle size^17^,^20^,^21^,^22^. The two ligands used here, thiolated (2-hydroxypropyl)-*β*-cyclodextrin (designated MUA-CD) and thiolated hyaluronic acid (designated *m*-HA), not only serve as capping agents during the formation of nanoscaled liquid-metal spheres but also play roles of drug-loading matrix and active targeting moiety, respectively. The final formulation (designated LM-NP/*L*) comprises three primary functional constituents: a CD-based drug-loading motif, the targeting ligand HA and an EGaIn core. The seven-membered sugar ring of the CD provides faithful loading sites for doxorubicin (Dox)^23^, a model broad-spectrum chemotherapeutic drug. The HA moiety supports active tumour targeting towards the receptors including the CD44 receptor, which is overexpressed on the cell surface of a broad variety of tumours, including human cervical cancer and breast cancer^24^. After intravenous injection, LM-NP/*L* is expected to accumulate at tumour sites as a result of passive^25^ and active targeting effects. Upon endocytosis, the LM-NPs/*L* are expected to fuse with each other in the mildly acidic endosome microenvironment^26^, leading to the dissociation of Dox-containing ligands and therefore promote drug release. The aggregates of fused LM-NPs/*L* are expected to degrade due to the synergistic effect of oxidative corrosion. Furthermore, the main degradation product, Ga (III), can serve as anticancer agent itself to reverse drug resistance in drug-resistant cancer cells^27^,^28^. Taken together, this liquid metal-based nanomedicine is transformable: (1) it can be simply generated from a bulk material; (2) fusion happens to promote drug release in an acidic cellular environment; and (3) it can be biodegraded to reverse drug resistance and avoid potential systemic toxicity.

## Results

### Preparation of transformable liquid-metal nanomedicine

To prepare the liquid metal nanocarriers, EGaIn was added to a centrifuge tube filled with an ethanolic solution of thiolate CD and thiolate HA (volume ratio of EGaIn: ethanol=1:150, Fig. 1a,b,e). After sonication, the largest particles precipitated within seconds, and the nanoscale colloids was removed from the tube and resuspended in water (Fig. 2a). Next, Dox-loaded LM-NP/*L* (LM-NP/Dox-*L*) was obtained by overnight incubation with triethanolamine-treated Dox. The mixture was washed to remove the excess Dox. The resulting LM-NP/Dox-*L* appeared as core–shell structured nanospheres with a diameter of ∼107 nm, which was consistent with the results determined by dynamic light scattering tests (Fig. 2b). The Dox-loading capacity could be tuned by adjusting the ratio between MUA-CD and *m*-HA. In a typical formulation used in the following investigations, the Dox-loading capacity was determined as 24% (Dox weight ratio of the total weight of nanoparticles). Interestingly, the addition of *m*-HA could significantly decrease the average particle size of product as well as enhance the systemic stability, which could be attributed to the negative charge of the anionic *m*-HA (Supplementary Table 1).

To study the acid-triggered conformational transformation and subsequently promoted drug release, the release profile of Dox from LM-NP/Dox-*L* (Fig. 2c) under neutral (1 × PBS buffer, pH 7.4) and acidic (1 × PBS buffer, pH 5.0) conditions were investigated through the dialysis method^29^. A burst release of loaded Dox was clearly observed within the first 30 min in the acidic buffer, significantly different from the release curve recorded in the neutral buffer. Similarly, paclitaxel (PTX), which shows insignificant solubility change in the pH range of 2.5–8 (ref. 30), was loaded to LM-NP/*L* as a model drug. As expected, the release profile of LM-NP/*L* loaded with PTX also presented a high release rate within the first 30 min in the acidic buffer (Supplementary Fig. 4). The acidic buffer caused 72% of the loaded PTX to release within 4 h, which was significantly higher than the 28% in the neutral environment. Dox-loaded gold nanoparticles (GNPs/Dox) with an average diameter of 100 nm, where Dox was included into the MUA-CD ligands with the same Dox-loading capacity as LM-NP/Dox-*L*, were also applied as a control. Compared with GNP/Dox, LM-NP/Dox-*L* displayed a more potent acid-promoted Dox release at pH 5.0 (Supplementary Fig. 5). This phenomenon could be attributed to fusion of LM-NP/*L* triggered by the disruption of the oxidized shell of LM-NP/*L*, and the subsequent dissociation of surface ligands. Next, the conformational change of LM-NP/Dox-*L* with up to 4 h acid treatment (pH 5.0) was visualized using transmission electron microscopy (TEM) imaging (Fig. 2d). The initial fusion behaviour was captured within the first 5 min after acid treatment, and the aggregates formed by fusion increased in size over time. These results were in good agreement with the significant increase in size distribution upon acid treatment as measured by dynamic laser scattering (Fig. 2e). We hypothesized that the acid attacked the oxidized layer^20^ on the surface of the LM-NP/*L* to trigger fusion of LM-NP/*L* (Supplementary Figs 8,9; Fig. 2f), which further led to the fusion of the liquid inner cores^31^. Furthermore, the degradation of LM-NP/Dox-*L* at pH 5.0 was clearly observed via the optical microscope (Fig. 2g) and TEM imaging (Fig. 2d) over time; while negligible difference was observed in the case of neutral buffer. The representative TEM image of LM-NPs/Dox-*L* immersed in acidic (pH 5.0) PBS buffer for 72 h showed evidence of degradation into the hollow polymeric shells dotted with shrunk metallic cores in the inner wall evidenced the occurrence of degradation. To further verify the degradation of LM-NPs, the change of Ga (III) ion concentration in PBS buffer over time was monitored by the inductively coupled plasma optical emission spectrometry. The Ga (III) concentration in the acidic buffer displayed a notable increase over time; while the degradation of LM-NPs in neutral solution was moderate (Fig. 2f). The degradation process of LM-NPs was then tracked and quantified by monitoring the change of light transmittance over time (Fig. 2h). The light transmittance of acidic LM-NP solution increased from 25 to 66%, which was in good agreement of the proposed degradation of LM-NPs in acidic environment. Of note, the main degradation product, Ga (III) ion, has been reported to reverse drug resistance in drug-resistant cancer cells^27^,^28^, which could potentially generate synergistic effects and prevent drug resistance.

Furthermore, the nature of the EGaIn metal core and the verified acid-triggered fusion might also open the possibility of utilizing these transformable nanospheres for imaging contrast enhancement. After acid treatment (12 h in 1 × PBS buffer, pH 5.0), the homogeneous contrast enhancement caused by LM-NPs was disrupted, replaced by heterogeneous contrast within the imaged area with higher contrast at the locations of fused particles and lower contrast at spots in absence of LM-NPs (Fig. 2i). The resulting changes in contrast were quantified by studying the grey value profiles of representative areas in X-ray images (Fig. 2i). The grey values of the imaging area were approximately constant before acid treatment, indicating homogeneous imaging contrast. The disruptions of the homogeneous imaging contrast after acid treatment were supported by the appearance of peaks in the grey value plot. Similar fusion of LM-NPs accumulated at tumour site would potentially lead to enhanced contrast of tumour tissue during X-ray imaging.

### Intracellular acid-triggered fusion and promoted Dox release

**Figure 1:**
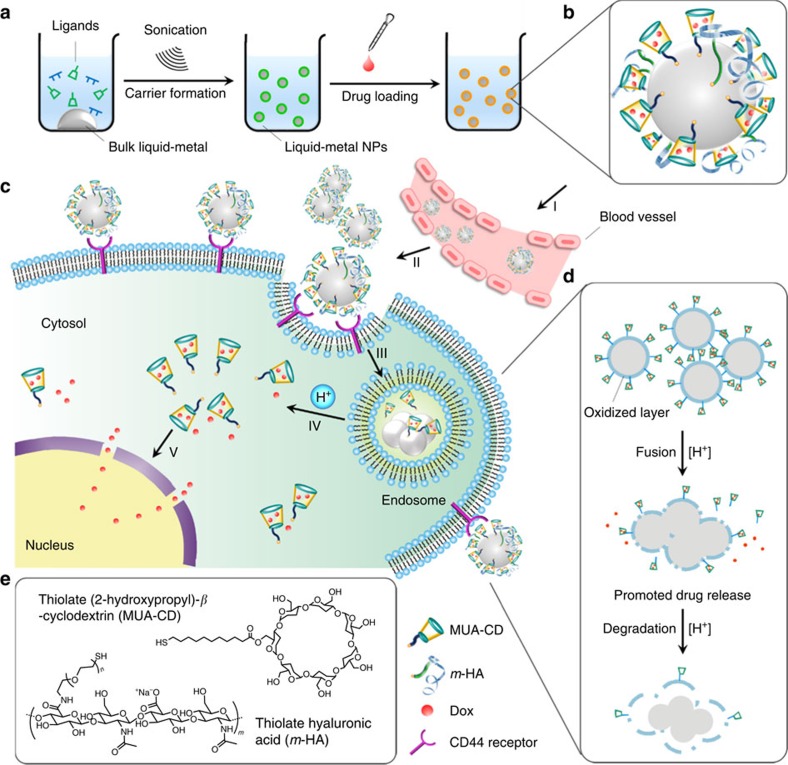
Schematic design of the transformable liquid-metal delivery system. (**a**) Preparation route of LM-NP/Dox-*L*. (**b**) The main components of LM-NP/Dox-*L*: thiolated CD with Dox, HA-based targeting motif and an EGaIn core. (**c**) pH-responsive delivery of Dox by LM-NP/Dox-*L* to the nuclei for the targeted cancer therapy. (I) accumulation of LM-NP/Dox-*L* at the tumour site through passive and active targeting; (II) specific binding to the overexpressed receptors on the tumour cells; (III) receptor-mediated endocytosis; (IV) acid-triggered fusion of LM-NP/Dox-*L* and endosomal/lysosomal escape of Dox-containing ligands; (V) accumulation of Dox in the nucleus. (**d**) Acid-triggered fusion and degradation process of LM-NP/Dox-*L*. (**e**) Chemical structures of MUA-CD and *m*-HA.

**Figure 2:**
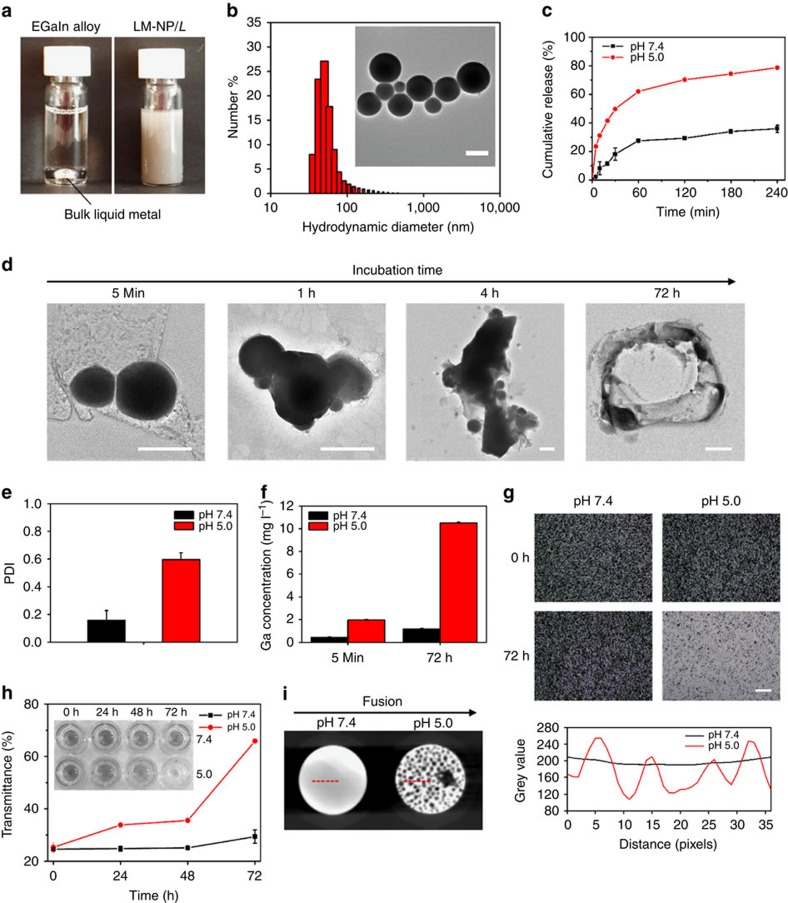
Characterization of liquid-metal nanospheres. (**a**) EGaIn was ultrasonically dispersed in ligand containing ethanol mixture (shown schematically in Fig. 1a). (**b**) The hydrodynamic size of LM-NP/Dox-*L* measured by dynamic light scattering. Inset: TEM image of LM-NP/Dox-*L*. Scale bar, 100 nm. (**c**) The release profiles of LM-NP/Dox-*L* at different pH levels. (**d**) Representative TEM images of LM-NP/Dox-*L* after different time immersed in acidic (pH 5.0) PBS buffer. Scale bars, 100 nm (for 5 min); 100 nm (for 1 h); 100 nm (for 4 h); 400 nm (for 72 h). (**e**) Polydispersity index (PDI) of LM-NP/Dox-*L* immersed in neutral (pH 7.4) and acidic (pH 5.0) PBS buffer. (**f**) Changes of metal ion concentration under neutral and acidic environments. (**g**) Optical images of LM-NPs before and after acid treatment. Scale bar, 40 μm. (**h**) Light transmittance change over time. (**i**) X-ray images (left) of LM-NPs immersed in neutral (pH 7.4) and acidic (pH 5.0) PBS buffer (particles were imaged after 12 h treatment). The images were taken in a 96-well plate. Red lines indicate the areas quantitatively analysed. Grey value analysis (right) of representative area in X-ray images of LM-NPs. Error bars indicated s.d. (*n*=3).

**Figure 3:**
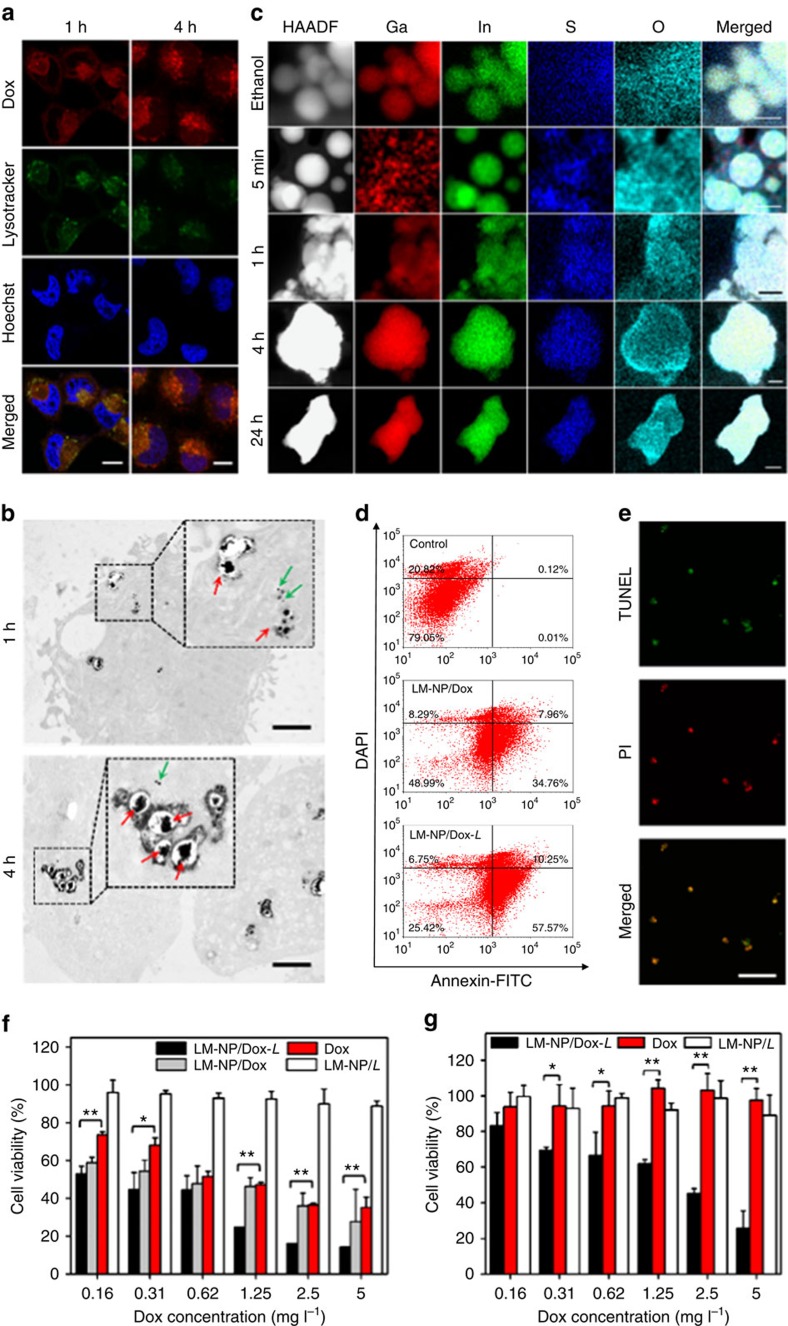
Intracellular interaction and drug release. (**a**) Intracellular delivery of LM-NP/Dox-*L* towards HeLa cells at different time points observed by confocal laser scanning microscopy. The cells were incubated with LM-NP/Dox-*L* at 37 °C for 1 and 4 h, respectively. The late endosomes and lysosomes were stained with LysoTracker Green, and the nuclei were stained with Hoechst 33342. Scale bar, 10 μm. (**b**) Representative TEM images of HeLa cells incubated with LM-NP/Dox-*L* for 1 and 4 h. Red arrows show fused nanospheres; green arrows show dispersion of single nanosphere in the cytosol. Scale bar, 2 μm. (**c**) Element mapping results of intracellular LM-NPs/Dox-*L* collected from HeLa cells after different incubation times. Scale bars, 100 nm (for 5 min); 100 nm (for 1 h); 100 nm (for 4 h); 200 nm (for 24 h). (**d**) Flow cytometric analysis of HeLa cell apoptosis induced by LM-NP/Dox-*L* for 12 h using the Annexin V-FITC/4,6-diamidino-2-phenylindole (DAPI) staining. (**e**) HeLa cell apoptosis induced by LM-NP/Dox-*L* for 20 h using the APO-BrdU TUNEL assay. (**f**) *In vitro* cytotoxicity of LM-NP/Dox and LM-NP/Dox-*L* on HeLa cells for 24 h. Error bars indicate s.d. (*n*=4). Note: the concentration of LM-NP/*L* is equal to the nanocarrier concentration of Dox-loaded formulations in each corresponding group; the error bars in **f** (group LM-NP/Dox-*L* at concentrations 1.25, 2.5 and 5 mg l^−1^) are small. (**g**) *In vitro* cytotoxicity of LM-NP/Dox-*L* on Dox-resistant HeLa cells for 24 h. Error bars indicate s.d. (*n*=4). Note: the concentration of LM-NP/*L* is equal to the nanocarrier concentration of Dox-loaded formulations in each corresponding group. *P<0.05, **P<0.01 compared with the Dox solution group (two-tailed Student's *t*-test).

The intracellular delivery of LM-NP/Dox-*L* into HeLa cells was further explored using the confocal laser scanning microscopy (Fig. 3a). The fluorescence of Dox was clearly observed in HeLa cells after 1 h of incubation with LM-NP/Dox-*L*, indicating the cellular internalization of the nanospheres. When the incubation time was prolonged to 4 h, Dox was highly localized within the nuclei of HeLa cells, as indicated by the magenta fluorescence (Fig. 3a). The cellular uptake of LM-NP/Dox-*L* displayed an energy-dependent manner, and LM-NP/Dox-*L* mainly entered HeLa cells via macropinocytosis (Supplementary Fig. 10). Next, the endocytosis process of LM-NP/Dox-*L* was also visualized by TEM imaging (Figs 1c,d and 3b). HeLa cells were treated with LM-NP/Dox-*L* for different time durations. After 1 h incubation, fusion of LM-NPs was clearly observed in the acidic endosomes, agreeing with the *in vitro* results discussed above. After 4 h incubation, more fusion events of LM-NPs were spotted, together with few single LM-NPs dispersed in the cytosol. Furthermore, we quantified and visualized the fusion process intracellularly by lysing the cells incubated with LM-NPs/Dox-*L* at different incubation time points. The intracellular Ga/In ion concentrations were then quantified by inductively coupled plasma mass spectrometry. Importantly, the structures formed via fusion were also further validated using TEM and analysed with the titan scanning/TEM at an atomic scale. Similar to the LM-NPs/Dox-*L* treated with acid (Fig. 2d), the initial fusion behaviour was captured immediately upon cellular uptake, followed by the formation of larger aggregates and the eventual degradation, which was in good agreement with the significant increase of intracellular Ga (III) ion concentration over incubation time (Supplementary Fig. 1). The element mapping results revealed the fusion of the polymeric shells during the conformational changes of LM-NP/*L* (Fig. 3c). The scattering of Ga signal after 5-min incubation indicated the fast disruption of the oxidized shell. After 1 h of incubation, the increase of S and O signals in the background suggested the dissociation of thiolated surface ligands. The time-of-flight secondary ion mass spectrometry was further utilized to analyse the surface of LM-NP/*L* and intracellular LM-NP/*L* collected by lysing the cells internalized with particles (Supplementary Fig. 2). The coexistence of thiols and oxides was well evidenced by the overlap of the signals of GaO^−^ and GaS^−^ in LM-NP/*L*. The increase of HS^−^ signal in the background after 5 min incubation visualized the dissociation of thiolated surface ligands, indicating the initial stage of fusion behaviour. The remarkable increase in the background HS^−^ signal and the significant decreases in the signals of GaO^−^ and GaS^−^ clearly indicated that the intracellular fusion behaviour of LM-NP/*L* was a result of the synergistic action of the dissociation of thiolated surface ligands and the disruption of oxides.

The Annexin V-FITC/4,6-diamidino-2-phenylindole apoptosis detection and the terminal deoxynucleotidyl transferase dUTP nick end labelling (TUNEL) assay were performed to compare the apoptosis-inducing capabilities of LM-NP/Dox-*L* and free Dox solution. Annexin V-FITC labels the phosphatidylserine translocated to the extracellular membrane upon the initiation of apoptosis^32^, while 4,6-diamidino-2-phenylindole has significantly higher staining efficiency with cells where the plasma membrane integrity has been compromised^33^. This combination allows the differentiation among the early apoptotic cells, the late apoptotic cells and the viable cells, which can be quantitatively determined by the flow cytometry. The total apoptotic ratio of HeLa cells incubated with LM-NP/Dox-*L* was 68%, which was significantly higher than 43% of HeLa cells incubated with LM-NP/Dox (Fig. 3d). In addition, the cells treated with LM-NP/Dox-*L* showed extensive apoptotic DNA fragmentation stained by the Alexa Fluor 488 as green fluorescence (Fig. 3e), substantiating that LM-NP/Dox-*L* is an effective intracellular delivery vehicle for enhanced apoptosis-inducing activity.

Next, the cytotoxicity of Dox-loaded nanospheres towards HeLa cells was evaluated with 3-(4, 5-dimethylthiazol-2-yl)-2, 5-diphenyltetrazolium bromide assay (Fig. 3f). Cell viability was dependent on both Dox concentration and incubation time, as expected. The half-maximal inhibitory concentration (IC_50_) of LM-NP/Dox and LM-NP/Dox-*L* towards HeLa cells for 24 h treatment were 0.81 and 0.23 mg l^−1^, respectively. LM-NP/Dox-*L* displayed higher cytotoxicity than free Dox solution (IC_50_=1.33 mg l^−1^), while blank LM-NPs/*L* without Dox showed insignificant cytotoxicity within the range of tested concentrations. At low Dox concentration (0.16 and 0.31 mg l^−1^), LM-NP/Dox also had higher cytotoxicity than free Dox solution. It was suggested that the acid-promoted release of Dox achieved by liquid metal nanospheres provided higher cytotoxic activity towards cancer cells. It should be noted that LM-NP/Dox-*L* showed significantly enhanced cytotoxicity (3.5-fold for 24 h treatment) compared with LM-NP/Dox towards HeLa cells that overexpress CD44 receptor^34^, which can be attributed to the HA-targeting moiety.

We further investigated *in vitro* cytotoxicity of LM-NP/Dox-*L* towards Dox-resistant HeLa cells. The results showed that LM-NP/Dox-*L* displayed significant cytotoxicity towards Dox-resistant HeLa cells (IC_50_=2.46 mg l^−1^), while free Dox solution did not show any obvious cytotoxicity even at the concentration of 5 mg l^−1^ (Fig. 3g). This result is encouraging and offers guideline for future applications of LM-NPs in the combination treatment of cancer drug resistance.

### Tumour targeting and antitumour efficacy of LM-NP/Dox-*L*

**Figure 4:**
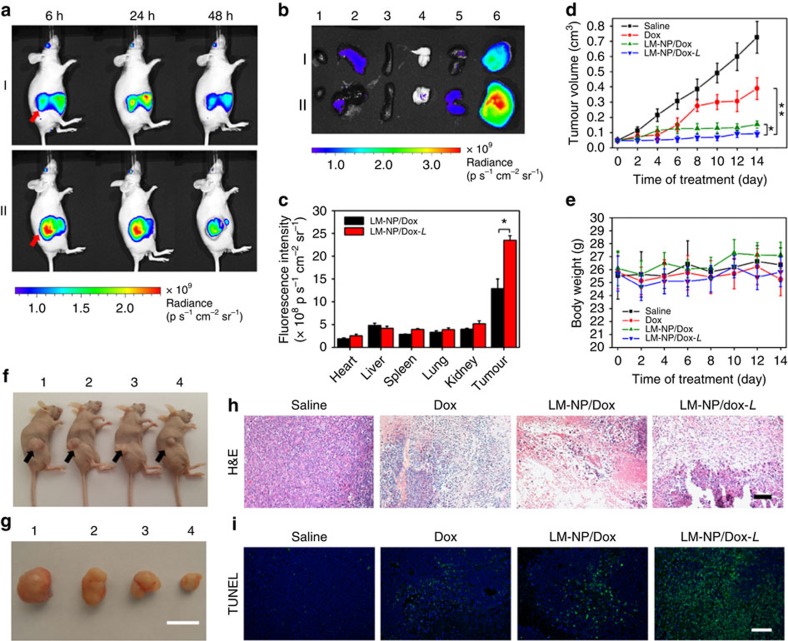
Tumour targetability and antitumour activity. (**a**) *In vivo* fluorescence imaging of the HeLa tumour-bearing nude mice at 6, 24 and 48 h after intravenous injection of Cy5.5-(LM-NP/Dox) (I) and Cy5.5-(LM-NP/Dox-*L*) (II) at Cy5.5 dose of 30 nmol kg^−1^. Arrows indicate the sites of tumours. (**b**) *Ex vivo* fluorescence imaging of the tumour and normal tissues collected from the HeLa tumour-bearing nude mice being killed at 48 h post injection. The numeric label for each organ is as follows: 1, heart; 2, liver; 3, spleen; 4, lung; 5, kidney; 6, tumour. (**c**) Region-of-interest analysis of fluorescent signals from the tumours and normal tissues. Error bars indicated s.d. (*n*=3). *P<0.05 (two-tailed Student's *t*-test). (**d**) The HeLa tumour growth curves after intravenous injection of different formulations of Dox at a dose of 2 mg kg^−1^. Error bars indicate s.d. (*n*=5). *P<0.05, **P<0.01 (two-tailed Student's *t*-test). (**e**) The body weight variation of HeLa tumour-bearing mice during treatment. Error bars indicate s.d. (*n*=5). (**f**) Representative images of the HeLa xenograft tumours of the mice after treatment with the studied Dox formulations at day 14. The numeric label for each mouse is as follows: 1, saline; 2, Dox; 3, LM-NP/Dox; 4, LM-NP/Dox-*L*. Arrows indicate the sites of tumours. (**g**) Representative images of HeLa xenograft tumours collected from the mice after treatment with different formulations at day 14. The numeric label for each tumour is as follows: 1, saline; 2, Dox; 3, LM-NP/Dox; 4, LM-NP/Dox-*L*. Scale bar, 1 cm. (**h**) Histological observation of the tumour tissues after treatment. The tumour sections were stained with haematoxylin and eosin (H&E). Scale bar, 100 μm. (**i**) Detection of apoptosis in the tumour tissues after treatment. The tumour sections were stained with fluorescein-dUTP (green) for apoptosis and Hoechst for the nuclei (blue). Scale bar, 50 μm.

To evaluate the tumour-targeting capability of LM-NP/Dox-*L* (Fig. 1c,d), Cy5.5-labelled LM-NP/Dox-*L* (Cy5.5-(LM-NP/Dox-*L*)) was administrated intravenously into the HeLa tumour-bearing mice. Within a short time period, Cy5.5-(LM-NP/Dox-*L*) presented a stronger fluorescence signal in the tumour region compared with Cy5.5-labelled LM-NP/Dox (Cy5.5-(LM-NP/Dox)) without the HA-targeting moiety (Fig. 4a). As time increased, elevated fluorescence signals of Cy5.5-(LM-NP/Dox-*L*) were observed at the tumour site as compared with the normal tissues within 48 h post injection, indicating a notable tumour-targeting effect of the nanospheres. After 48 h post injection, the mice were killed, and the tumours as well as normal tissues were collected for *ex vivo* imaging study. The intensity of fluorescence signal of Cy5.5-(LM-NP/Dox-*L*) at tumour site was significantly higher than that of Cy5.5-(LM-NP/Dox) (Fig. 4b). On the basis of the quantitative region-of-interest analysis, the fluorescence intensity of Cy5.5-(LM-NP/Dox-*L*) at the tumour site was 1.82-fold that of Cy5.5-(LM-NP/Dox) (Fig. 4c). In addition, the fluorescence signal at the tumour site was three times higher than that in the liver or kidney (Fig. 4b,c). The potent tumour-targeting capability of LM-NP/Dox-*L* can be attributed to the combination of an enhanced permeability and retention effect and active targeting mechanisms.

The time-dependent biodistribution of LM-NPs/*L* was further quantified by inductively coupled plasma mass spectrometry. HeLa tumour-bearing nude mice were killed at 6, 24 and 48 h after intravenous injection of LM-NPs/Dox-*L* for tissue collection. The results further substantiated the potent tumour-targeting capability of LM-NPs/Dox-*L* (Supplementary Fig. 11). The pharmacokinetics of LM-NP/Dox-*L* administered intravenously into mice was evaluated by quantitatively monitoring the Dox concentration in blood plasma (Supplementary Fig. 12). The elimination half-life (*t*_1/2_), the mean residence time and the area under the curve depicting the plasma drug concentration versus time were significantly higher than those of the free Dox solution, suggesting the capability of LM-NP/Dox-*L* to maintain a high drug concentration during a prolonged systemic circulation. The antitumour efficacy of LM-NP/Dox-*L* was further assessed in the HeLa tumour-bearing xenograft mice. Different Dox formulations displayed significant tumour inhibition effects compared with saline as a negative control after successive intravenous administration into the HeLa tumour-bearing mice (Fig. 4d, f, g). Both LM-NP/Dox and LM-NP/Dox-*L* displayed remarkably higher inhibition efficacy towards HeLa tumour growth than the free Dox solution, which primarily resulted from the tumour-targeting capability of these nanospheres. More importantly, a noticeable difference in tumour volume inhibition between LM-NP/Dox-*L* and LM-NP/Dox was observed (Fig. 4d,f,g), further demonstrating the enhanced active targeting capability of LM-NP/Dox-*L*. No significant change of mice body weights was observed during the treatment of LM-NP/Dox-*L* (Fig. 4e). The histological images using haematoxylin and eosin staining showed that a massive cancer cell remission occurred in the tumour tissue (Fig. 4h) after LM-NP/Dox-*L* administration, with no obvious pathological abnormalities in normal organs, including cardiomyopathy, the major toxic effect of Dox cancer treatment (Supplementary Fig. 13)^35^. Moreover, the images obtained using the *in situ* TUNEL assay showed the highest level of cell apoptosis in the tumour collected from the mice treated with LM-NP/Dox-*L* (Fig. 4i), indicating that the supreme tumour inhibition activity was attributable in part to the increased apoptosis induced by LM-NP/Dox-*L*. The anticancer behaviour of LM-NPs/*L* was also explored in tumour-bearing mice, where no significant anticancer effects were observed after intravenous injection of blank LM-NPs/*L* (Supplementary Fig. 14). Collectively, these results verified that HA-conjugated LM-NP/Dox-*L* efficiently accumulated at the tumour site, indicating effective receptor-mediated intracellular transport, and thereby achieved the optimal antitumour efficancy *in vivo*.

### Toxicology evaluation of LM-NP/Dox-*L*

**Figure 5:**
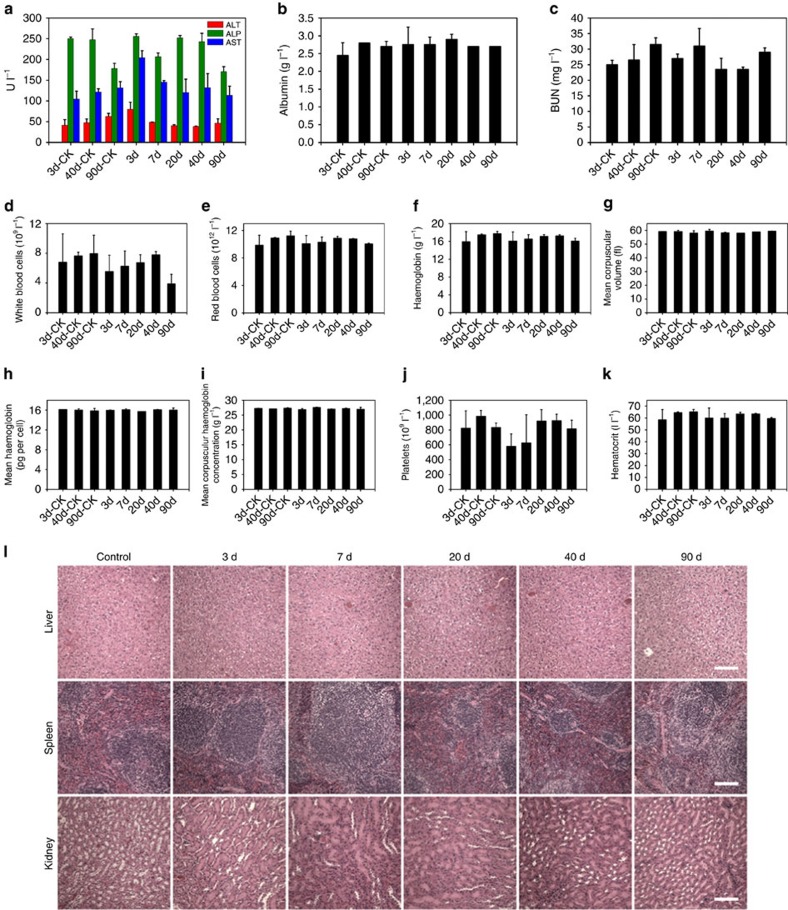
Toxicology evaluation. Blood biochemistry and haematology data of female Balb/c mice treated with LM-NPs/*L* at the dose of 45 mg kg^−1^ (total nanocarrier dose used in antitumuor efficacy study) at 3, 7, 20, 40 and 90 d: (**a**) alanine aminotransferase (ALT), alkaline phosphatase (ALP) and aspartate aminotransferase (AST) levels in the blood at different time points after LM-NPs/*L* treatment. (**b**,**c**) Time-course changes of albumin concentration and blood urea nitrogen (BUN). (**d-k**) Time-course changes of white blood cells (**d**), red blood cells (**e**), platelets (**f**), haemoglobin (**g**), mean corpuscular volume (**h**), mean corpuscular haemoglobin (**i**), mean corpuscular haemoglobin concentration (**j**) and haematocrit (**k**) from control mice (CK) and LM-NPs/*L*-treated mice. Error bars indicate s.d. (*n*=3). (**l**) Histology evaluation of the major organs (liver, spleen and kidney) collected from the control untreated mice and LM-NPs/*L*-injected mice at different time points post injection. Scale bars, 100 μm. d, days.

The toxicology of LM-NP/*L* to female Balb/c mice was investigated over 3 months (Fig. 5). The female Balb/c mice injected with LM-NP/*L* (45 mg kg^−1^) were killed at 3, 7, 20, 40 and 90 days post injection for blood collection. The levels of important liver function markers, including the alanine aminotransferase, aspartate aminotransferase, alkaline phosphatase and the albumin concentration were all within reference ranges^36^,^37^, indicating that no obvious hepatic toxicity was induced by LM-NP/*L* treatment (Fig. 5a,b). As an indicator of the kidney functions, the urea levels in the blood of treated mice were also within the normal range (Fig. 5c). For the haematological assessment, we selected white blood cells, red blood cells, haemoglobin, mean corpuscular volume, mean corpuscular haemoglobin, mean corpuscular haemoglobin concentration, platelet count and haematocrit (Fig. 5d–k). Although a vibration of platelets concentration was observed on day 3, the value itself still fell into a reference normal range^36^. In addition, a steady recovery in platelets level was observed over time. The platelets level fully recovered on day 20, with no significant difference compared with that of the age control group. All of the above indices in the LM-NP/*L*-treated groups were normal compared with the control groups. We also performed necropsy of the sacrificed mice, and no noticeable organ damage was observed (Fig. 5l). Tissues collected from the heart, brain and muscle were also investigated, and no obvious tissue injury was observed (Supplementary Fig. 15). Taken together, we concluded that LM-NP/*L* displayed no obvious toxicity at the treatment dose, which was highly encouraging for the application of LM-NP/*L* as nanomedicine.

The single-dose, acute toxicity of LM-NP/*L* and LM-NP/Dox-*L* to female Balb/c mice was investigated by identifying the maximum tolerated dose (MTD). The estimated MTD of LM-NP/*L* was determined to be 700 mg kg^−1^, suggesting low acute toxicity. The estimated MTD of LM-NP/Dox-*L* was found to be 55 mg kg^−1^ (Dox dose), 1.75-fold higher than that of free Dox solution. In addition, no obvious increase in IgE production was observed in LM-NP/*L*-injected mice, revealing no sign of allergic reactions (Supplementary Fig. 16).

Moreover, we have also monitored the time-dependent excretion to study the *in vivo* metabolism of LM-NPs/*L* (Supplementary Fig. 17). The results suggested that the clearance of LM-NPs/*L* was possibly through both fecal and renal excretions. The excreted concentrations of both gallium and indium were steadily decreased over time.

## Discussion

We have developed a new liquid metal-based drug delivery platform for anticancer therapy. The formulation can be easily formed and tailored via ligand-mediated self-assembly using facile ultrasonication. The resulting liquid-metal nanospheres are able to fuse for promoting drug release and eventually degrade under a mild acidic environment. After fusing, these nanospheres also display a contrast enhancing capability when imaged by X-ray, suggesting potential as a theranostic reagent. Systematic investigation of toxicology of LM-NP/*L* revealed no obvious toxicity at the treatment dose, favouring its biomedical applications. Although there were no obvious signs of toxicity in these studies, we will further evaluate the systemic toxicity of the developed formulations, as well as other promising candidates made from different liquid metal, such as pure gallium. Moreover, considering other physical properties of liquid metal, including capability of dissolving iron or other metals, mechanical flexibility towards tissues and electrical conductivity, many new materials and scaffolds integrated with liquid metal for drug delivery and tissue engineering can be envisioned.

## Methods

### Materials

All chemicals were purchased from Sigma-Aldrich (St Louis, MO, USA) unless otherwise stated. Thiol–polyethylene glycol–amine (HS-PEG-NH_2_) with the PEG molecular weight of 1,000 was purchased from Sigma-Aldrich and Nanocs Inc. (New York, NY, USA). Dox hydrochloride was purchased from BIOTANG Inc. (Lexington, MA, USA).

### Preparation and characterization

For a typical LM-NP/*L* preparation, a small amount of EGaIn (80 μl) was added to a centrifuge tube (50 ml), which was filled to a total volume of 12 ml with ethanol containing 4 mg thiolate CD and 0.5 mg thiolate HA. After sonication, the largest particles precipitated within seconds, and the slurry was removed from the vial. The remained solution was further purified by mild centrifugation (1,000 r.p.m.) to remove relatively large particles. The resulting nanospheres were collected via centrifugation and resuspended in PBS buffer. To prepare LM-NP, EGaIn (80 μl) was added to a centrifuge tube (50 ml), which was filled to a total volume of 12 ml with ethanol containing 4 mg thiolate CD. After sonication, the largest particles precipitated within seconds, and the slurry was removed from the vial. The remained solution was further purified by mild centrifugation (1,000 r.p.m.) to remove relatively large particles. The resulting nanospheres were collected via centrifugation and resuspended in PBS buffer. Cy5.5-labelled nanospheres were prepared by adding Cy5.5-conjugated HS-PEG-NH_2_ (molecular weight: 1,000) into the ethanol containing mixture. For loading of Dox, 1 ml of LM-NP/L was incubated with 100 μl of triethanolamine-treated Dox in dimethyl sulfoxide (5 mg ml^−1^) with stirring at room temperature overnight. The resulting Dox-loaded LM-NP/*L* was extensively washed with PBS via centrifugation to remove the excess Dox. LM-NP/Dox, LM-NP/Rho and LM-NP/Rho-*L* were prepared following the same procedure. For TEM characterization, LM-NP/Dox was cast onto a TEM copper grid (300 mesh; Ted Pella). After drying in air, the sample was observed by TEM (JEM-2000FX, Hitachi) operating at 200 kV.

### *In vitro* acid-triggered Dox and PTX release

LM-NP/Dox and LM-NP/Dox-*L* (0.5 ml) were added into a dialysis tube (10K MWCO; Slide-A-Lyzer, Thermo Scientific) and dialysed against 14 ml of the PBS buffer solution at pH 5.0 or 7.4, and gently shaken at 37 °C in a shaker (New Brunswick Scientific) at 100 r.p.m. At predetermined time intervals, the total buffer solution was replaced with 14 ml of fresh buffer solution with the same pH. The fluorescence intensity of Dox released was measured at 596 nm with an excitation wavelength of 480 nm using a microplate reader (Infinite M200 PRO, Tecan). PTX release study was conducted following the same procedure. The concentration of PTX was determined by high-performance liquid chromatography (Hewlett Packard 1100).

### Cell culture

HeLa cells were obtained from Tissue Culture Facility of UNC Lineberger Comprehensive Cancer Center and cultured in DMEM with 10% (v:v) fetal bovine serum, 100 U ml^−l^ penicillin and 100 μg ml^−l^ streptomycin in an incubator (Thermo Scientific) at 37 °C under an atmosphere of 5% CO_2_ and 90% relative humidity. The cells were sub-cultivated approximately every 3 days at 80% confluence using 0.25% (w:v) trypsin at a split ratio of 1:5.

### Intracellular trafficking

HeLa cells (1 × 10^5^ cells per well) were seeded in a confocal microscopy dish (MatTek). After culture for 24 h, the cells were incubated with LM-NP/Dox-*L* (2 μM Dox concentration) at 37 °C for 1 h and 4 h, respectively, and then washed twice using PBS at 4 °C. Subsequently, the cells were stained with LyosTracker Green (50 nM) (Life Technologies) at 37 °C for 30 min and Hoechst 33342 (1 μg ml^−1^; Life Technologies) at 37 °C for 10 min. Finally, the cells were washed using PBS twice at 4 °C and immediately observed using confocal laser scanning microscopy (LSM 710, Zeiss).

### *In vitro* cytotoxicity

HeLa cells (1 × 10^4^ cells per well) were seeded in the 96-well plates. After culture for 24 h, the cells were exposed to the Dox solution and Dox-loaded LM-NPs with different concentrations of Dox for 24 h, followed by adding 20 μl of the 3-(4, 5-dimethylthiazol-2-yl)-2, 5-diphenyltetrazolium bromide solution (5 mg ml^−l^). After 4 h of incubation, the medium was removed, and the cells were mixed with 150 μl of dimethyl sulphoxide. The absorbance was measured at a test wavelength of 570 nm and a reference wavelength of 630 nm by a microplate reader (Infinite M200 PRO, Tecan).

### Apoptosis assay

Apoptosis of HeLa cells was detected using the APO-BrdU TUNEL Assay Kit (Life Technologies) and Annexin V-FITC Apoptosis Detection Kit (BD Biosciences). The cells (1 × 10^5^ cells per well) were seeded in the six-well plates. After culture for 48 h, the cells were incubated with Dox-loaded LM-NPs for 12 h (Annexin V-FITC) or 20 h (TUNEL). The subsequent procedures were performed in accordance with the manufacturer's protocol. For Annexin V-FITC apoptosis detection, the cells were analysed by flow cytometry (BD FACSCalibur), while for the TUNEL assay the cells were observed by fluorescence microscope (IX71, Olympus).

### Animals and tumour xenograft models

All animals were treated in accordance with the Guide for Care and Use of Laboratory Animals, approved by the Institutional Animal Care and Use Committee (IACUC) of North Carolina State University. To set-up the tumour xenograft model, the female nude mice (6 weeks, J:NU, The Jackson Laboratory) were subcutaneously inoculated in the back with 1 × 10^7^ HeLa cells. The tumour size was monitored by a vernier caliper and the tumour volume (*V*) was calculated as *V*=*L* × *W*^2^/2, where *L* and *W* were the length and width of the tumour, respectively.

### *In vivo* imaging study

The animal study protocol was approved by the Institutional Animal Care and Use Committee at North Carolina State University and University of North Carolina at Chapel Hill. When the tumours reached to 200–400 mm^3^, the mice were intravenously injected by Cy5.5-(LM-NP/Dox) and Cy5.5-(LM-NP/Dox-*L*) at Cy5.5 dose of 30 nmol kg^−1^. Images were taken on the IVIS Lumina imaging system (Caliper, USA) at 6, 24 and 48 h post injection^29^. After the 48-h scanning, the mice were killed. The tumours as well as major organs were collected, weighed and subjected for *ex vivo* imaging. Region-of-interests were circled around the organs, and the fluorescence intensities were analysed by Living Image Software.

### *In vivo* antitumour efficacy

The tumour-bearing mice were weighed and randomly divided into different groups when the tumour volume reached to 50 mm^3^. From day 0, the mice were intravenously injected with Dox solution (2 mg kg^−1^), LM-NP/Dox (2 mg kg^−1^), LM-NP/Dox-*L* (2 mg kg^−1^) and saline as a negative control every other day for 12 days, and meanwhile the tumour size was measured. At day 14, the mice were killed, and the tumour as well as the heart were collected, weighed, washed with saline thrice and fixed in the 10% neutral-buffered formalin. For the haematoxylin and eosin staining, the formalin-fixed tumours and hearts were embedded in paraffin blocks and visualized by optical microscope (DM5500B, Leica). For the TUNEL apoptosis staining, the fixed tumour sections were stained by the *In Situ* Cell Death Detection Kit (Roche Applied Science) according to the manufacturer's protocol. Hoechst 33342 was used for nuclear counterstaining. The stained tumour slides were observed by fluorescence microscope (IX71, Olympus).

## Additional information

**How to cite this article:** Lu, Y. *et al.* Transformable liquid-metal nanomedicine. *Nat. Commun.* 6:10066 doi: 10.1038/ncomms10066 (2015).

## Supplementary Material

Supplementary Information Supplementary Figures 1-17, Supplementary Table 1, Supplementary Methods and Supplementary References
