# Supplementary material for: Transformable liquid-metal nanomedicine
Source: Nat Commun. 2015 Dec 2;6:10066. doi: 10.1038/ncomms10066 (PMC4686762; doi:10.1038/ncomms10066)
Supplement: Supplementary Information — Supplementary Figures 1-17, Supplementary Table 1, Supplementary Methods and Supplementary References [file ncomms10066-s1.pdf]

Supplementary Figures

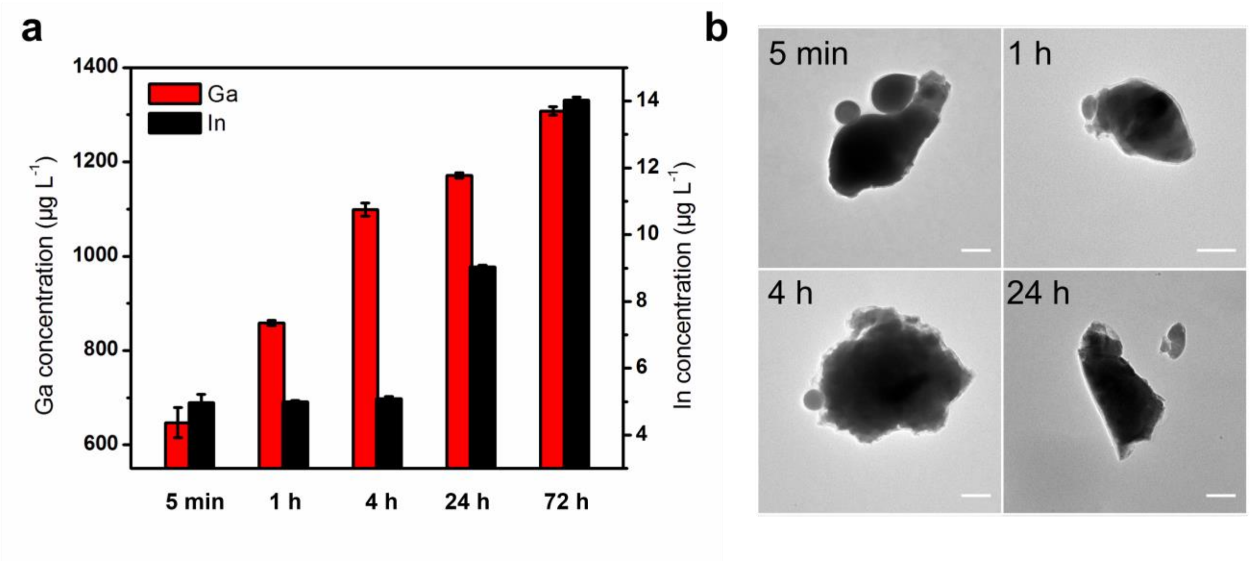

Supplementary Figure 1| Intracellular fusion and degradation. (a) Changes of intracellular metal ion concentrations. Intracellular ion concentrations were normalized by total protein concentration. (b) Representative TEM images of intracellular LM-NPs/Dox-*L* collected from HeLa cells after different incubation time. Scale bars: 100 nm.

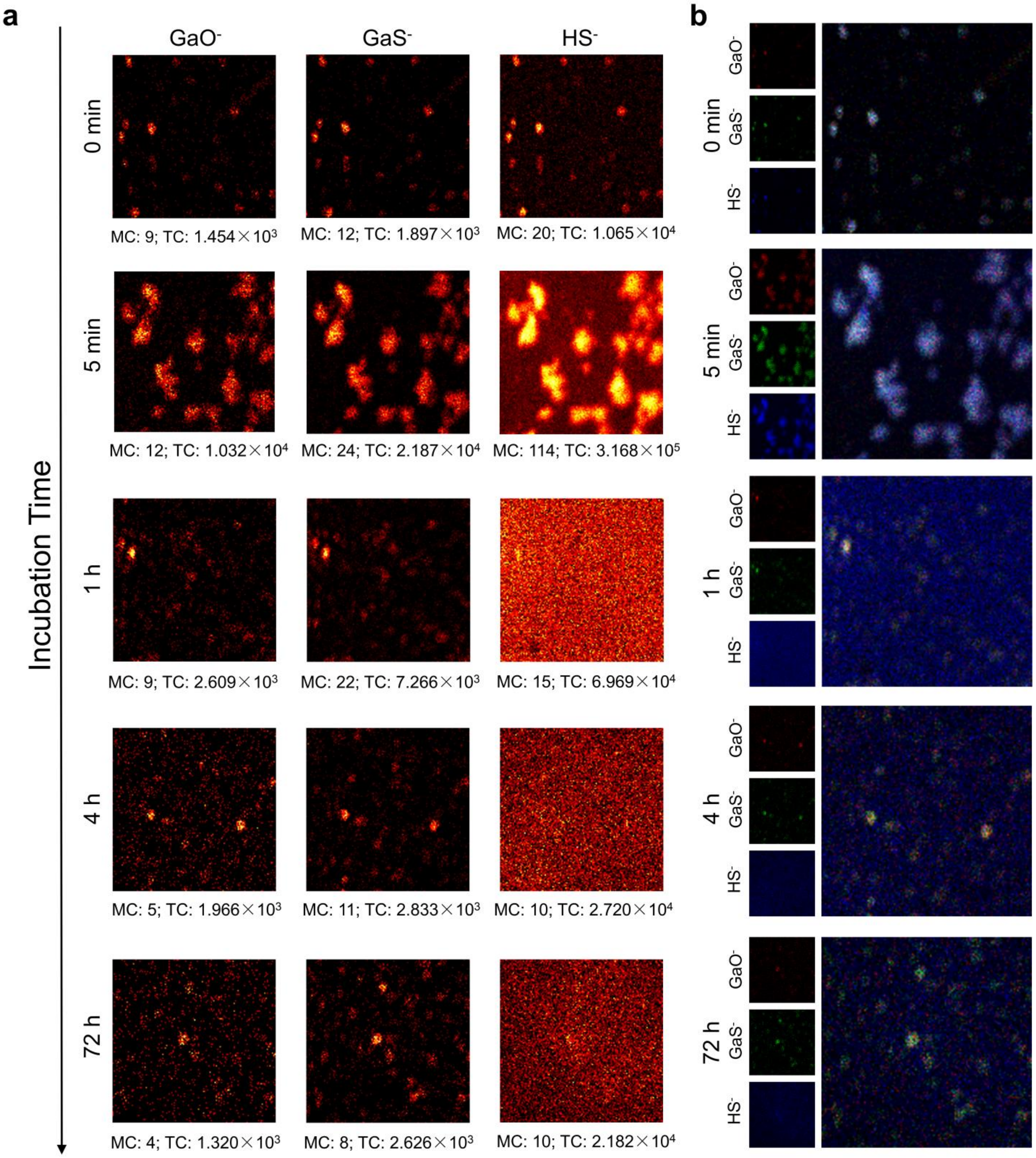

Supplementary Figure 2| Changes of surface chemistry of LM-NP/*L* during intracellular fusion and degradation monitored by the Time-of-Flight Secondary Ion Mass Spectrometry (TOF-SIMS). (a) Signal intensity distributions of GaO^-, GaS^- and HS^- in LM-NP/*L* collected at different incubation time. Each image represents an area of 500 μm×500 μm. MC: number of secondary ions in the brightest pixel. TC: total counts of detected secondary ions. (b) Overlay of the signals of GaO^- (red), GaS^- (green) and HS^- (blue) in LM-NP/*L* collected at different incubation time.

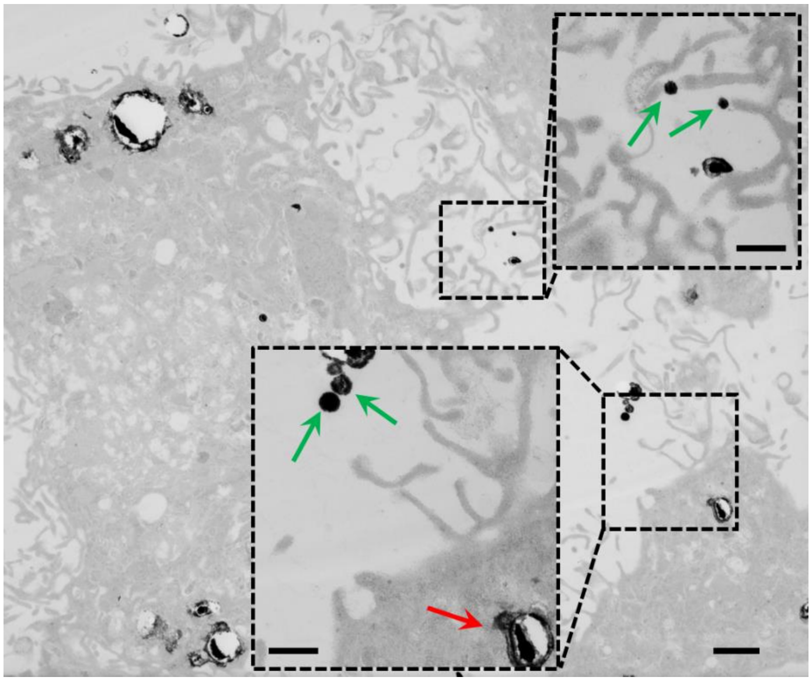

Supplementary Figure 3| Representative TEM image of HeLa cells incubated with LM-NP/Dox-*L*. Red arrows show fused nanospheres after endocytosis; green arrows show single nanospheres before endocytosis. Scale bar: 1 μm. Inset: zoom in of locations of interest, scale bars: 200 nm.

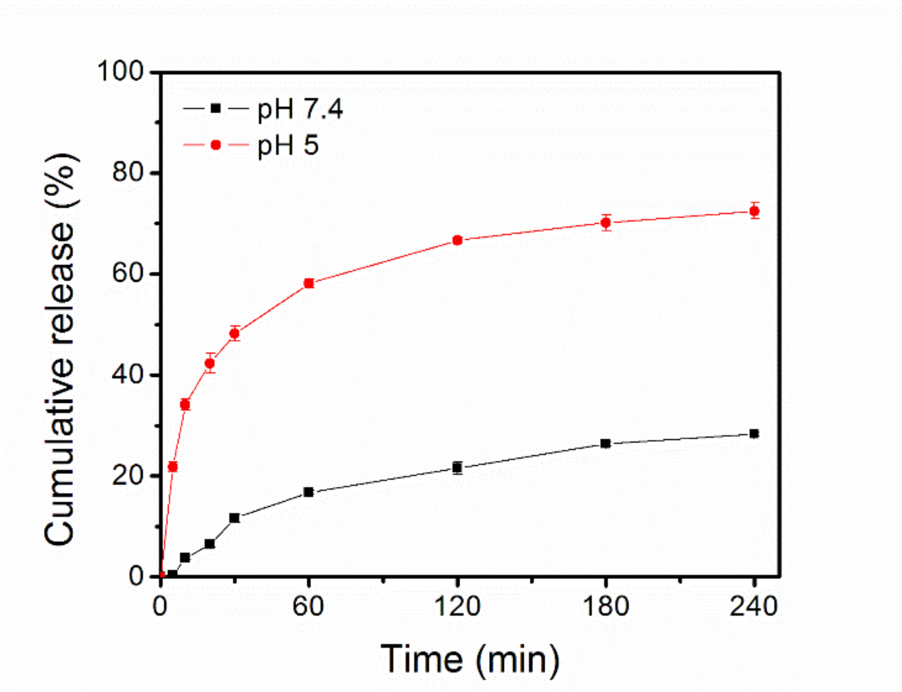

Supplementary Figure 4| The release profiles of LM-NP/PTX-*L* at different pH levels. Error bars indicate s.d. (n=3).

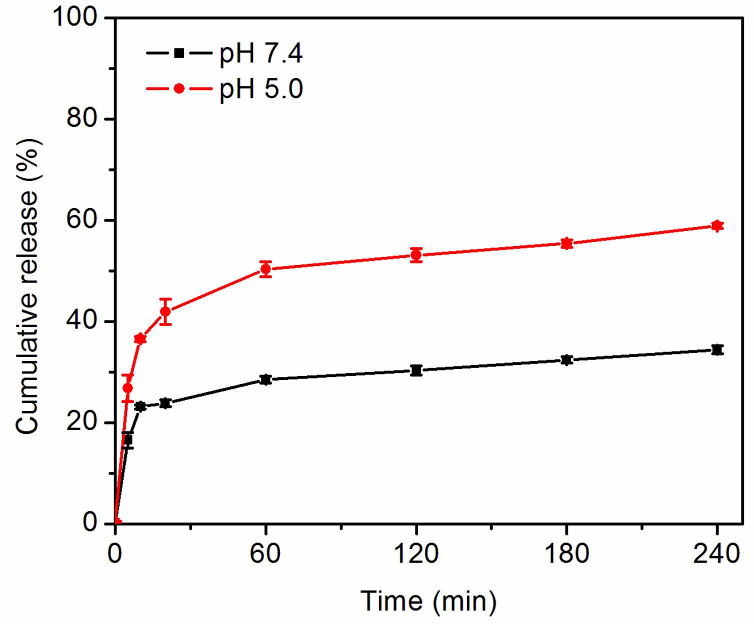

Supplementary Figure 5| The release profiles of GNP/Dox at different pH levels. Error bars indicate s.d. (n=3).

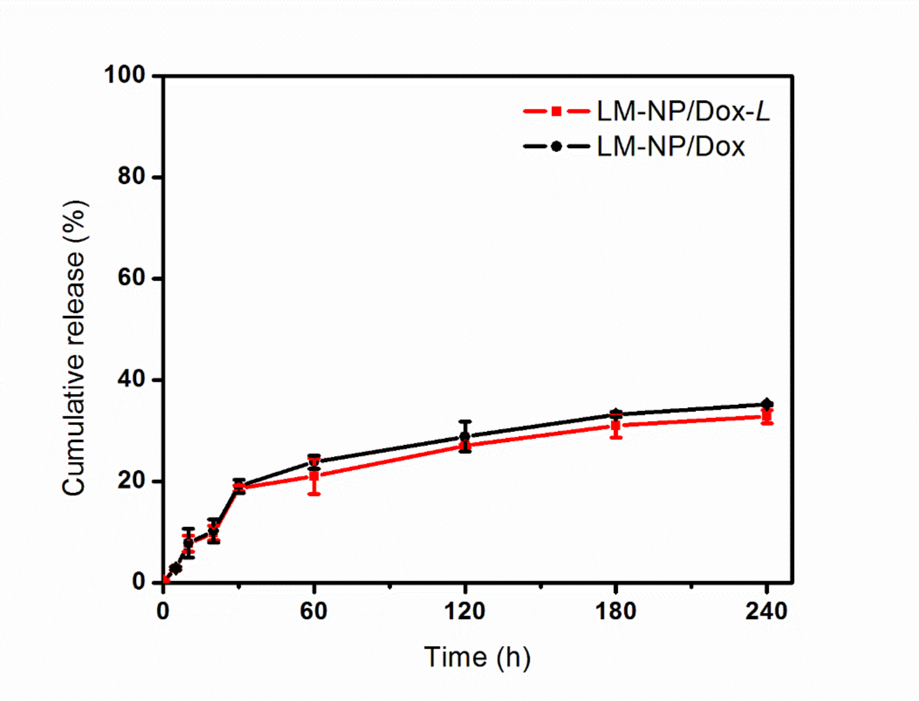

Supplementary Figure 6| The release profiles of LM-NP/Dox and LM-NP/Dox-*L* in 10% fetal bovine serum (PBS).

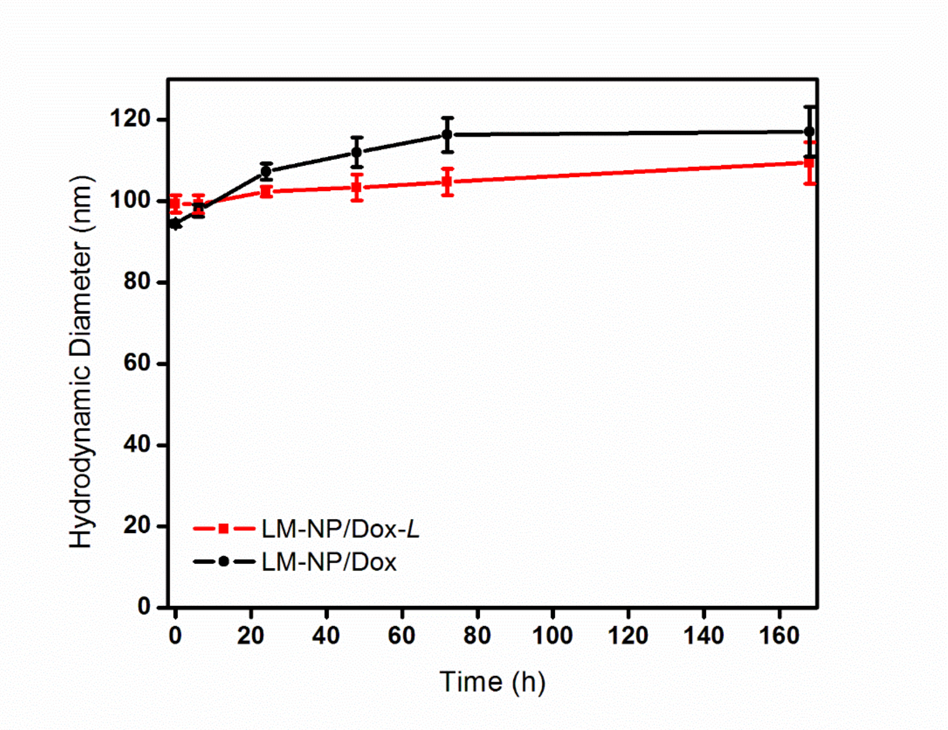

Supplementary Figure 7| Hydrodynamic size of LM-NP/Dox and LM-NP/Dox-*L* in 10% fetal bovine serum (PBS).

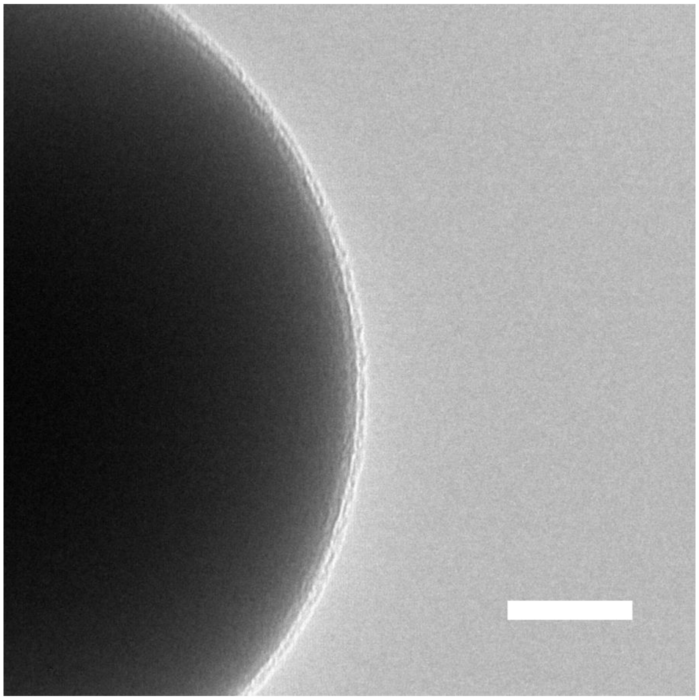

Supplementary Figure 8| High-resolution transmission electron microscopy (HRTEM) image of the outer shells of EGaIn particle. Scale bar is 20 nm.

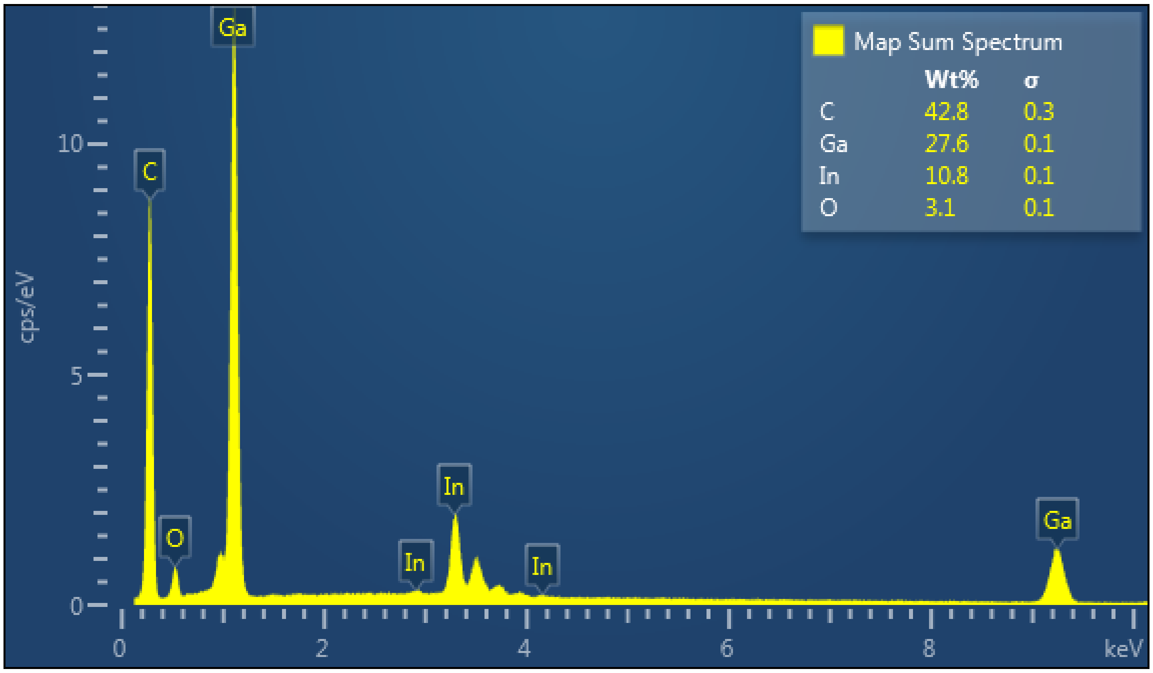

Supplementary Figure 9| EDS (energy-dispersive X-ray spectroscopy) spectrum of EGaIn particles.

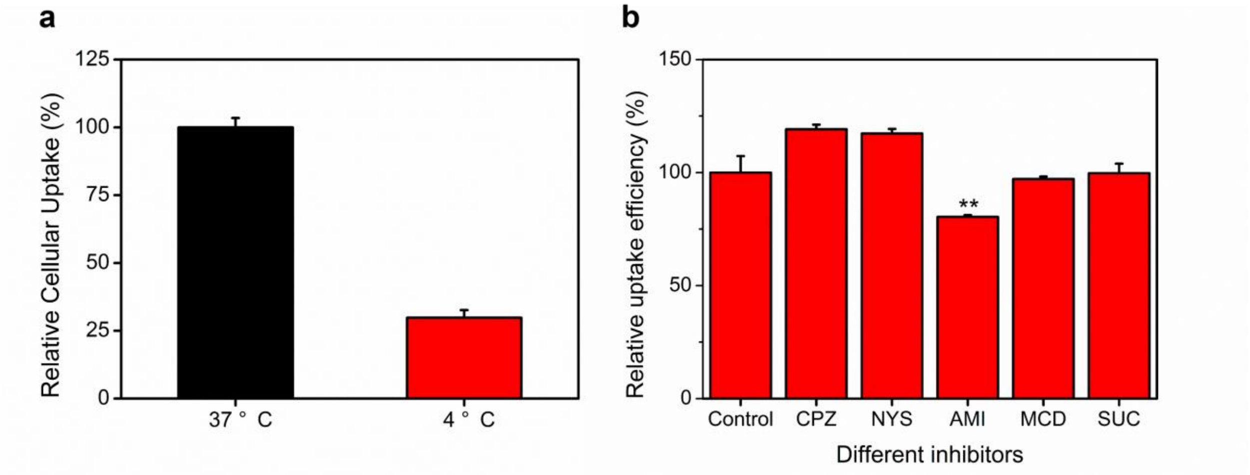

Supplementary Figure 10| Study of cellular uptake mechanism. (a) Relative uptake efficiency of LM-NP/Dox-*L* on HeLa cells at 4 °C and 37 °C. (b) Relative uptake efficiency of LM-NP/Dox-*L* on HeLa cells in the presence of various endocytosis inhibitors. Error bars indicate s.d. (n = 3). $^{*}P < 0.05$, $^{**}P < 0.01$ compared with the control group (two-tailed Student's t-test). Inhibitor of clathrin-mediated endocytosis: sucrose (SUC) and chlorpromazine (CPZ); inhibitor of caveolin-mediated endocytosis: nystatin (NYS); inhibitor of macropinocytosis: amiloride (AMI); inhibitor of lipid raft: methyl-*β*-cyclodextrin (MCD). Compared with the cellular uptake of LM-NP/Dox-*L* at 37°C, the significant decrease in uptake of LM-NP/Dox-*L* at 4°C indicated an energy dependent uptake mechanism. Compared with the cellular uptake of LM-NP/Dox-*L* without inhibitors as a control, the significant decrease and increase in uptake of LM-NP/Dox-*L* with inhibitors confirmed the corresponding endocytosis pathways of the nanoparticles.

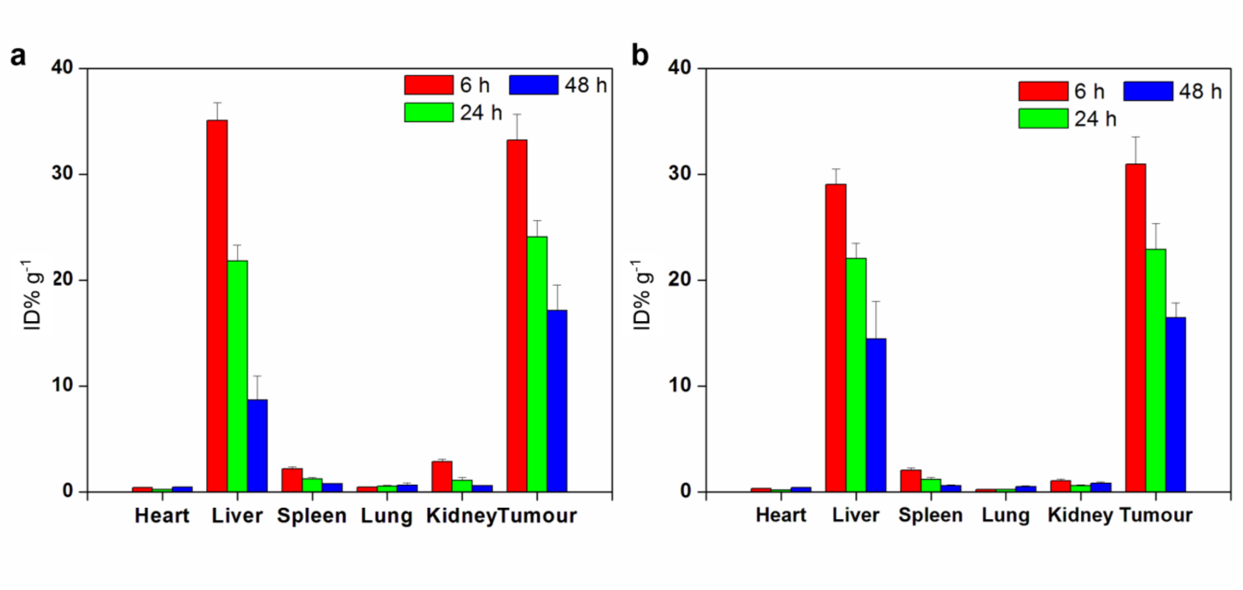

Supplementary Figure 11| Time-dependent biodistribution of LM-NP/Dox-*L* in Hela tumour-bearing nude mice at 1 h, 6 h, 24 h and 48 h after intravenous injection of LM-NP/Dox-*L*. (a) Time-dependent biodistribution of LM-NP/Dox-*L* determined by gallium concentration. (b) Time-dependent biodistribution of LM-NP/Dox-*L* determined by indium concentration. Error bars indicate s.d. (n=3).

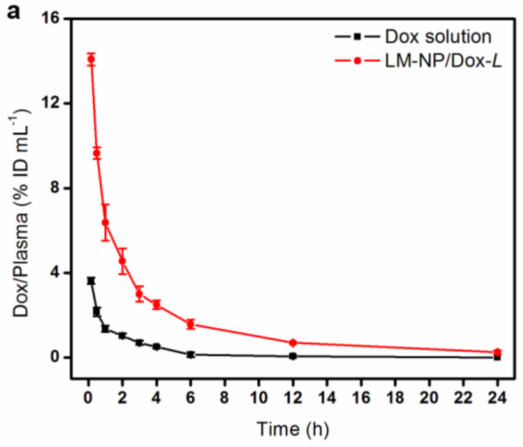

b

Parameter	Dox	LM-NP/Dox-*L*
C_{max}[a] (μg mL^{-1})	1.45±0.06	5.63±0.12**
AUC[b] (μg (mL×h)$^{-1}$)	2.75±0.12	16.55±0.94**
$t_{1/2}$[c] (h)	1.11±0.02	6.30±1.22**
MRT[d] (h)	2.80±0.61	6.38±0.68**

[a]Maximum Dox concentration in plasma

[b]Area under the plasma Dox concentration versus time curves

[c]Elimination half-life

[d]Mean residence time

***P*<0.01 versus Dox

Supplementary Figure 12| Pharmacokinetics of Dox after intravenous injection of the Dox solution and LM-NP/Dox-*L* into mice at Dox dose of 2 mg kg^{-1}. (a) The plasma concentration versus time curves of the Dox solution and LM-NP/Dox-*L*. Dox/Plasma (% ID mL^{-1}) is the ratio of the Dox amount in the plasma to the total injected dose (ID). Error bars indicate s.d. (n = 3). (b) Pharmacokinetic parameters of the Dox solution and LM-NP/Dox-*L*. Data are means ± s.d. (n = 3). *P < 0.05, **P < 0.01 compared with the Dox solution group (two-tailed Student's *t*-test).

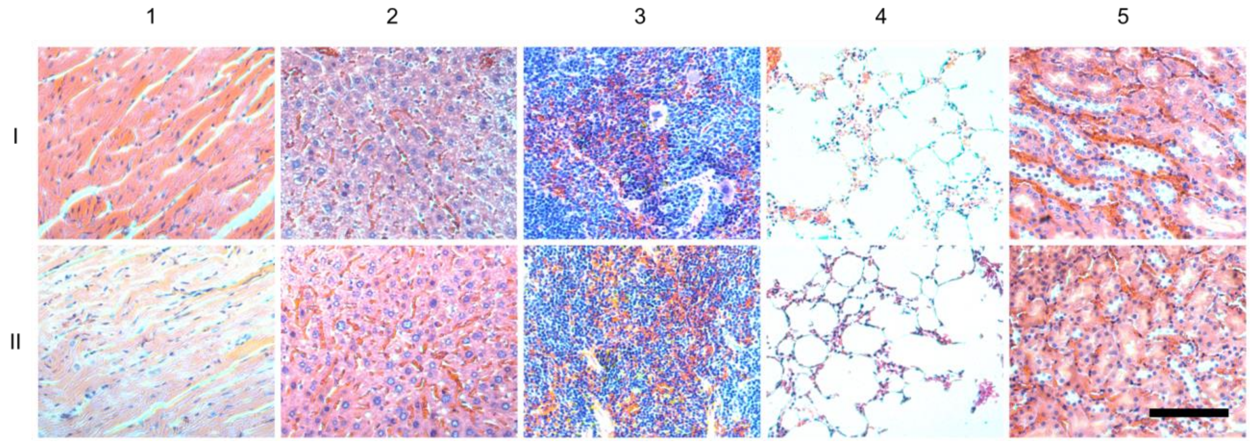

Supplementary Figure 13| Histological observation of the normal organs (1. Heart, 2. Liver, 3. Spleen, 4, Lung and 5. Kidney) collected from the mice after treatment with PBS (I) and LM-NP/Dox-*L* (II) at Day 14. Scale bar is 100 μm.

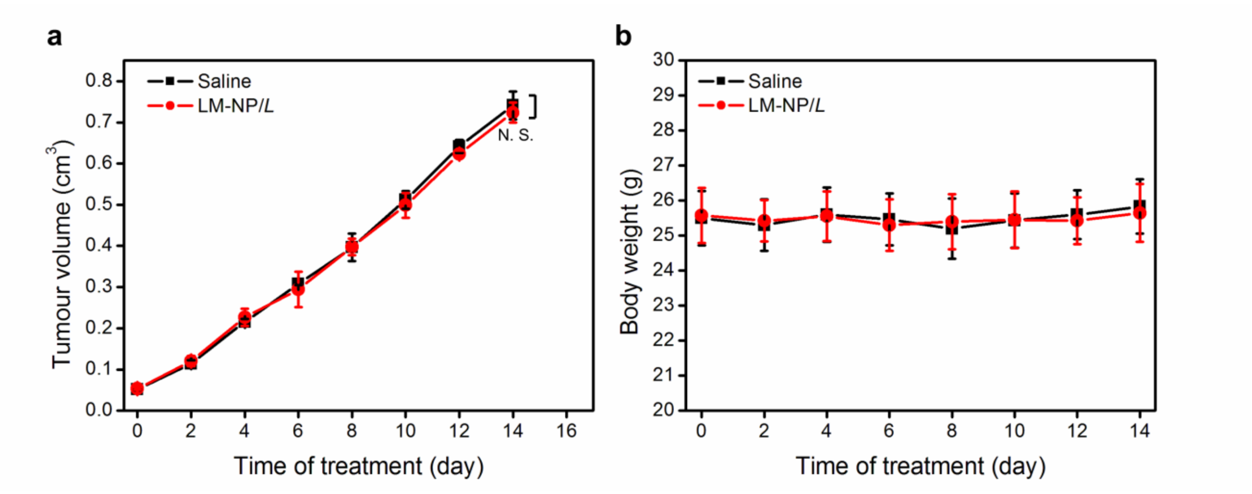

Supplementary Figure 14| Antitumour activity of blank LM-NP/*L*. (a) The tumour growth curves after intravenous injection of blank LM-NP/*L*. Error bars indicate s.d. (n=5). N.S. (no significance) indicates *P* > 0.05, LM-NP/*L* compared with saline (two-tailed Student's *t*-test). (b) The body weight variation of tumour-bearing mice during treatment. Error bars indicate s.d. (n=5).

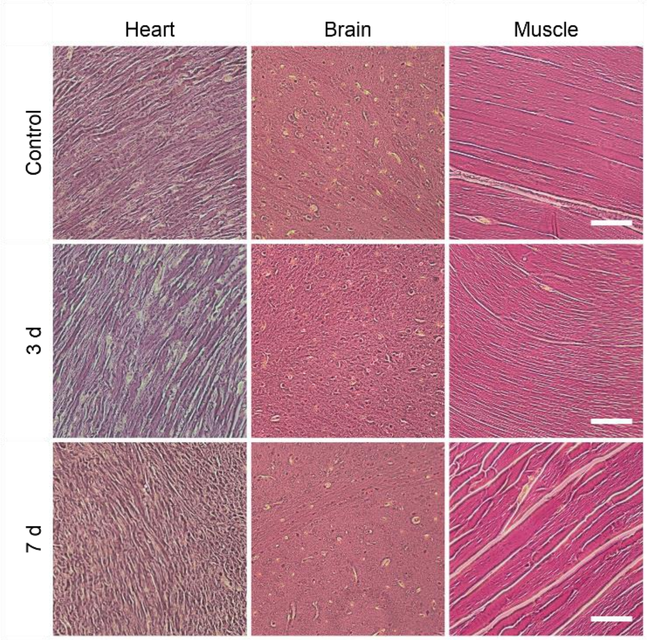

Supplementary Figure 15| Histology evaluation of organs (heart, brain and muscle) collected from the control untreated mice and LM-NPs/*L* injected mice (dose: 45 mg kg^{-1}) at day 3 and day 7 post injection. Scale bars: 100 μm.

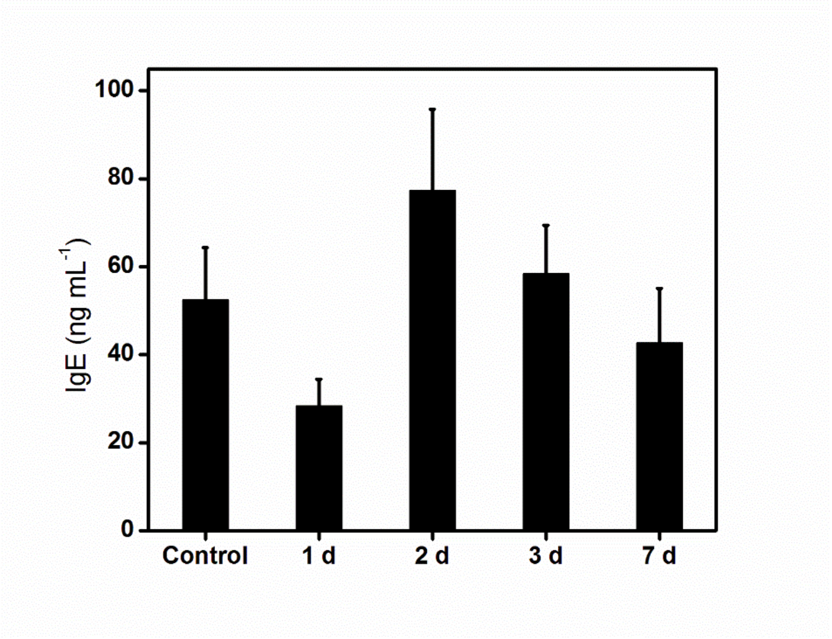

Supplementary Figure 16| Serum IgE levels in female Balb/c mice injected with PBS and LM-NPs/*L* (dose: 45 mg kg^{-1}). Error bars indicate s.d. (n=3).

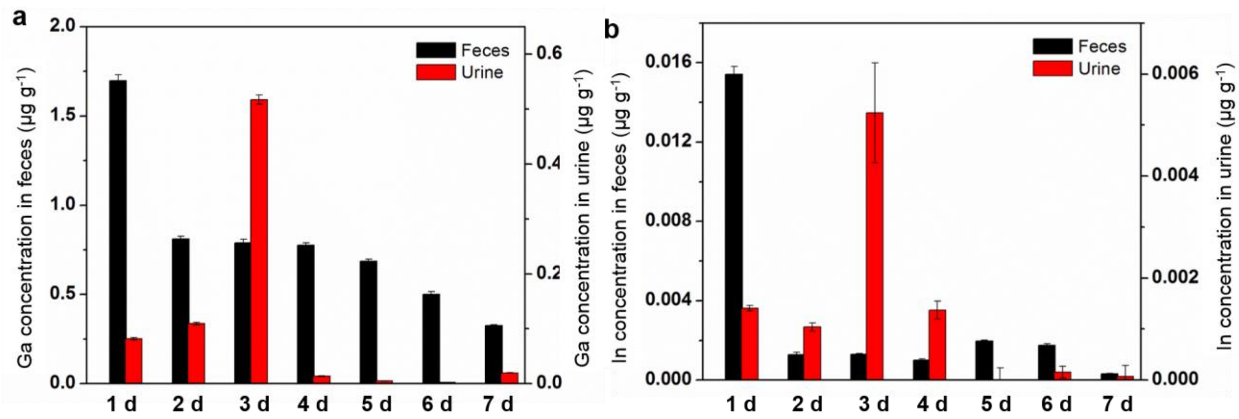

Supplementary Figure 17| LM-NPs/*L* levels in feces and urine determined by gallium (a) and indium (b) in the first week after injection. Error bars indicate s.d. (n=3).

Supplementary Table

Supplementary Table 1| The zeta-potentials and averaged hydrodynamic sizes of LM-NPs.

	LM-NP	LM-NP/*L*	LM-NP/Dox-*L*
Zeta-potential (mV)	-14.1±0.8	-24.7±2.1	-25.1±1.1
Hydrodynamic size (nm)	125	104	107

Supplementary Methods

Instruments. The particle size and Zeta potential were measured by the Zetasizer (Nano ZS, Malvern). The fluorescence intensity was measured by a microplate reader (Infinite M200 PRO, Tecan). Transmission electron microscopy (TEM) images of LM-NPs were obtained on a JEOL 2000FX Scanning Transmission Electron Microscope (STEM). High-resolution transmission electron microscopy (HRTEM) image of oxidized shell was obtained on a JEOL 2010 field emission ultra-high resolution STEM. TEM images of cell sections were obtained on a JEOL 1200EX TEM. Element mapping of single particles was obtained on a TITAN STEM. EDS (energy-dispersive X-ray spectroscopy) spectrum was obtained on a FEI Titan 80-300 probe aberration corrected STEM with SuperX EDS system. X-ray imaging was performed on GE eXplore CT120 micro-CT scanner. Laser scanning confocal microscopy images were obtained with the Carl Zeiss LSM 710 Laser Scanning Microscope.

Synthesis and characterization of MUA-CD. (2-hydroxypropyl)-*β*-cyclodextrin (CD, 0.279 g), 11-mercaptoundecanoic acid (MUA, 0.048 g) and 4-Dimethylaminopyridine (DMAP, 0.002 g) were dissolved in 20 mL of dry *N,N'*-dimethylformamide (DMF), followed by the addition of *N,N'*-Dicyclohexylcarbodiimide (DCC, 0.041 g). The reaction was allowed to proceed at room temperature overnight. The reaction mixture was first purified by filtration. After rotary evaporation, the resulting MUA-CD was characterized by ^{1}H NMR (Varian Gemini 2300).

MUA-CD: ^{1}H NMR (DMSO d_6, 300 MHz, δ ppm): 1.01, 1.30, 1.51-1.72, 3.20-3.89, 4.39-5.05, 5.51-6.02.

Synthesis and characterization of *m*-HA. Sodium hyaluronate (HA, 1.08g, 3 mg/mL) was dissolved in 45 mL of DI water. HS-PEG-NH_2 was dissolved in 45 mL of DI water. Solutions were combined, EDC and NHS were added as powder, and system pH was adjusted to 5.5 ± 0.3 with NaOH/HCl solutions. After 45 min, reaction pH was increased to and maintained at 7.3 ± 0.3. The reaction was allowed to proceed at room temperature for 4 days. After dialysis against DI water for 48 h, the white powder (*m*-HA) was obtained by means of freeze-drying, and characterized by ^{1}H NMR (Varian Gemini 2300).

m-HA: ^{1}H NMR (D_2O, 300 MHz, δ ppm): 1.82, 2.60-2.68, 3.07-3.43, 3.46-3.66.

Determination of drug loading capacity. The loading capacity (LC) of LM-NP/Dox-*L* was calculated as: LC=(A-B)/C, where A was the expected encapsulated amount of Dox, B was the free amount of Dox in the collection solution and C was the total weight of nanoparticles.

Preparation of Dox loaded gold nanoparticles. First, gold nanoparticles (diameter: 100 nm, purchased) were functionalized with MUA *via* ligand exchange reaction. The resulting MUA functionalized gold nanoparticles were then dispersed in DMF, followed by the addition of CD in presence of DCC and DMAP. The reaction was allowed overnight. After the synthesis, the CD functionalized gold nanoparticles (GNPs) were isolated through centrifugation, washed with DI water and filtered using PVDF 45-μm filter (Fisherbrand) to remove excess DCC. For loading of Dox, 1 mL of GNP was incubated with 100 μL of triethanolamine (TEA)-treated Dox in dimethyl sulfoxide (DMSO) (5 mg mL^{-1}) with stirring at room temperature overnight. The resulting Dox-loaded GNP/Dox was extensively washed with PBS *via* centrifugation to remove the excess Dox. The surface functionalization degree was fine tuned to achieve the same LC as LM-NP/Dox-*L* (24%).

Determination of endocytosis pathways. HeLa cells (1×10^5 per well) were seeded in the 6-well plates and cultured for 48 h. In order to investigate whether the cellular uptake of LM-NP/Dox-*L* was energy dependent, the cells were incubated with LM-NP/Dox-*L* at a Dox concentration of 2 μM at 4 °C and 37 °C, respectively. To determine the endocytosis pathway, the cells were pre-incubated with several specific inhibitors for various kinds of endocytosis [inhibitor of clathrin-mediated endocytosis: sucrose (SUC, 450 mM)[1] and chlorpromazine (CPZ, 10 μM)[2]; inhibitor of caveolin-mediated endocytosis: nystatin (NYS, 25 μg mL^{-1})[3]; inhibitor of micropinocytosis: amiloride (AMI, 1mM)[4]; inhibitor of lipid raft: methyl-*β*-cyclodextrin (MCD, 3 mM)[5]] at 37 °C, respectively. Afterward, the cells were incubated with LM-NP/Dox-*L* at a Dox concentration of 2 μM in the presence of inhibitors for additional 2 h. After washing the cells with 4 °C PBS twice, the fluorescence intensity of Dox in the cells and the cell proteins were measured, respectively.

Intracellular fusion and degradation. HeLa cells (1×10^5 per well) were seeded in the 6-well plates and cultured for 48 h, followed by incubation with LM-NP/Dox-*L*. At different time points, the cells were thoroughly washed and lysed. After centrifugation, the metal ion concentrations and total protein amounts in supernatants were determined by ICP-MS and BCA assay, respectively. The pellets were carefully washed with DI water and collected for TEM imaging and element analysis.

Determination of maximum tolerated dose. The maximum tolerated dose (MTD) was defined as the highest dose that does not cause major adverse reactions in mice over 10 days post single dose intravenous injection[6]. In this study, major adverse reactions were considered to be immediate death, impaired mobility or irregular breathing that could not be recovered within a day, over 10% weight loss over continuous days, or histological evidence of organ toxicity.

Supplementary References

1 Hsieh, C., Brown, S., Derleth, C. & Mackie, K. Internalization and Recycling of the CB1 Cannabinoid Receptor. *J. Neurochem.* **73**, 493-501 (1999).

2 Mo, R., Jiang, T., DiSanto, R., Tai, W. & Gu, Z. ATP-triggered anticancer drug delivery. *Nature Commun.* **5**, 3364 (2014).

3 Singh, R. D. *et al.* Selective Caveolin-1–dependent Endocytosis of Glycosphingolipids. *Mol. Biol. Cell* **14**, 3254-3265 (2003).

4 Sallusto, F., Cella, M., Danieli, C. & Lanzavecchia, A. Dendritic cells use macropinocytosis and the mannose receptor to concentrate macromolecules in the major histocompatibility complex class II compartment: downregulation by cytokines and bacterial products. *J. Exp. Med.* **182**, 389-400 (1995).

5 Zidovetzki, R. & Levitan, I. Use of cyclodextrins to manipulate plasma membrane cholesterol content: evidence, misconceptions and control strategies. *Biochim. Biophys. Acta* **1768**, 1311-1324 (2007).

6 Yu, T., Greish, K., McGill, L. D., Ray, A. & Ghandehari, H. Influence of geometry, porosity, and surface characteristics of silica nanoparticles on acute toxicity: their vasculature effect and tolerance threshold. *ACS Nano* **6**, 2289-2301 (2012).
